# Adjustment experiences of adolescents living with well-controlled type 1 diabetes using closed-loop technology

**DOI:** 10.3389/fcdhc.2024.1445972

**Published:** 2024-10-17

**Authors:** Sylvia Kruger, Elmari Deacon, Esmé van Rensburg, David Segal

**Affiliations:** ^1^ Compres Research Focus Area, Faculty of Health Sciences, North-West University, Potchefstroom, South Africa; ^2^ Optentia Research Focus Area, North-West University, Vanderbijlpark, South Africa

**Keywords:** adjustment, adolescents, closed-loop technology, interpretative phenomenological analysis, type 1 diabetes

## Abstract

**Aim:**

This study aimed to obtain an in-depth understanding of the experiences of adolescents with well-controlled type 1 diabetes who were adjusting to closed-loop technology.

**Method:**

Interpretative Phenomenological Analysis (IPA) was conducted. Five participants (aged 15–18) were recruited from the Centre for Diabetes and Endocrinology in Parktown, South Africa, to participate in semi-structured interviews about their experiences of adjusting to closed-loop technology.

**Results:**

Five superordinate themes emerged (1): learning to trust the technology (2), making diabetes visible (3), building a relationship with diabetes (4), empowering support networks, and (5) transformative positive outcomes. The findings demonstrate that closed-loop technology positively impacts the adjustment to living with type 1 diabetes. However, as highlighted by all participants, the individual’s engagement and management are crucial. Based on the adolescents’ experiences, interventions should focus on psychological factors.

## Introduction

Type 1 diabetes is a chronic autoimmune condition in which a person living with diabetes is unable to produce sufficient insulin or does not produce it at all (1, 2). In 2022, the global population of individuals living with type 1 diabetes reached 8.75 million. Of the global population of people living with type 1 diabetes, 1.52 million were under the age of 20 (3). If left untreated, damage to many of the body’s organs can occur, leading to life-threatening complications such as kidney damage, eye disease, and cardiovascular disease (2). However, these complications can be avoided by maintaining glucose levels within a target range (4).

Diabetes technology refers to the use of a broad array of medical devices designed to monitor and manage diabetes. These devices are utilised by individuals with diabetes in a home setting. This umbrella term encompasses a wide range of tools, from basic glucose meters and insulin pens to more advanced systems, such as continuous glucose monitors (CGMs) and insulin pumps. Closed-loop technology, viewed as the pinnacle of diabetes management innovation, falls under this increasingly expansive umbrella. Closed-loop technology is characterised by real-time glucose-responsive insulin administration, in which insulin delivery is partially automated by adjusting it on an insulin pump based on glucose readings from a CGM (5). A CGM device provides glycaemic readings and the direction of upward or downward readings approximately every five minutes (6). Within the realm of insulin pumps, closed-loop systems stand out because they integrate CGM data to partially automate insulin delivery by adjusting the insulin delivery on the pump based on glucose readings from a CGM (5).

Closed-loop technology is a valuable tool for people with diabetes and holds promise for improving glycaemic control (7). The reported benefits of closed-loop technology include improvement in glycaemic control, reduction of hypoglycaemia and diabetic ketoacidosis, increased time in range (TIR), improved sleep, psychosocial benefits such as peace of mind, reduced anxiety about hypo- and hyperglycaemia, an improved sense of safety (8, 9) and improved quality of life (6). Utilising data from closed-loop technology also enables the integration of CGM data into treatment modalities and decisions (9). Thus, closed-loop technology has the advantage of providing a person living with diabetes and healthcare providers with more insight into glucose trends and patterns (9).

Despite these advantages, closed-loop systems do not work well for every person living with diabetes, and the monetary cost of closed-loop technology is high (1). Some people do not experience improved control, and many discontinue insulin pump usage (6). During the adolescent years, discontinuity rates tend to be higher (10), and despite the benefits thereof, young people often struggle with the use of long-term diabetes technology (11). Adolescence is a transitional developmental stage characterised by biological, psychological, and social changes and has been conceptualised as a period of transition from dependence to independence (12). Key developmental tasks during this period include developing identity and autonomy, becoming socially responsible, and acquiring a set of values to guide behaviour (13), amidst significant biological and hormonal changes occurring during this period (14). The arrival of adolescence signifies new challenges for diabetes management, as indicated by the deterioration in adherence to the treatment regimen and deteriorating glucose control (15). Those with type 1 diabetes in this age group are often described as having deteriorating glycaemic control, poor treatment adherence, and an increased risk of psychological conditions (16, 17). It is estimated that only 21% of people between the ages of 13 and 19 meet the recommended glucose targets (18). The prospect of technology in achieving medical benefits largely depends on the engagement and use of technology by individuals with diabetes (15, 19). This highlights the complex interplay between technology, engagement with healthcare professionals, and psychological factors in diabetes management, which is the central theme of our investigation. Both medical factors (such as interactions with healthcare professionals and advancements in diabetes medication) and psychological factors should be considered to effectively integrate diabetes technology into the daily lives of individuals with diabetes (6).

Although there is some understanding of the adjustment process to closed-loop technology, there is a notable gap in research regarding the specific challenges faced by adolescents. Additionally, there is a need for more targeted exploration into how to support and educate individuals with diabetes, particularly in relation to specific medical technologies, to maximize their benefits (6). To address this gap, it would be beneficial to examine existing research on the use of diabetes technology more broadly and apply those insights to the adolescent population and specific technologies.

Numerous studies e.g (20, 21). have focused on the reasons why individuals fail to achieve diabetes control within the recommended targets. However, little is known about the experiences of those who meet these targets (22). Further investigation is needed to understand adolescents’ experiences of adjusting to living with type 1 diabetes and using closed-loop technology. Clarifying adolescents’ adjustment experiences can provide a basis for psychological and medical interventions to improve glycaemic control (17), as a more patient-centred approach to diabetes technology is needed (6). In South Africa, the management of type 1 diabetes in adolescents is compounded by cultural perceptions, economic disparities, and the availability of healthcare resources. These factors not only affect access to advanced diabetes management technologies but also influence psychological and social adjustment processes for adolescents. In South Africa, closed-loop technology is available only in the private sector.

This study focuses on adolescents who have successfully controlled their condition and integrated closed-loop technology into their lives, offering valuable insights that contrast with much of the existing research, which often emphasises rejection, non-adoption, and poor use of such technologies. Additionally, the Unified Theory of Acceptance and Use of Technology (UTAUT) was used during the discussion phase to frame and interpret the findings. The UTAUT is a theoretical model that aims to explain the adoption and use of technology (6, 23), emphasising the critical role of an individual’s beliefs, attitudes, and perceptions when making decisions about using technology (22). The UTAUT has been adopted and used in the research field of medical devices and technology (24).

To explore in detail how participants made sense of their personal experiences, IPA (25) was deemed suitable. The authors developed the following research question: What are the lived experiences of adolescents living with type 1 diabetes who successfully adjusted to closed-loop technology? The research question reflects a focused enquiry into the interplay between technology, psychology, and diabetes management.

## Method

### Study design

Interpretative Phenomenological Analysis (IPA) suited the research aim well, as it focuses on obtaining an in-depth understanding of the lived experience of a homogenous sample (25, 26). This method is committed to exploring how people make sense of their major life experiences as experts based on their personal insights (25). IPA is considered particularly valuable for gaining deeper insights into the experiences of those living with closed-loop technology, and it proves to be highly useful in the context of health psychology (27).

### Study population

We conceptualised ‘successful management’ as not just achieving medical targets but also navigating and integrating technology into daily life in a manner that supports overall well-being. Individuals with well-controlled diabetes are likely to employ effective strategies for using and incorporating closed-loop systems. Well-controlled diabetes was defined medically as adolescents using closed-loop technology and achieving a Haemoglobin A1c (HbA1c) of < 7% and a TIR of > 70% (referred to as ‘well-controlled’ diabetes) (28, 29). HbA1c is a test that indicates the average glycaemic control over a three-month period. This indicates the risk of developing complications (30).

### Sampling and recruitment

A non-probability sample was used, and participants were recruited using homogenous purposive sampling (31). Purposive sampling was used, as information-rich participants for whom the research question was relevant would maximise the understanding of the phenomenon (32). IPA aims to understand experiences in depth; hence, a small sample was selected in line with the IPA literature (27). A relatively homogenous sample is recommended by the IPA literature (25); therefore, the researchers recruited five participants between the ages of 15 and 18 years from the Centre for Diabetes and Endocrinology (CDE), Parktown, South Africa, who met the inclusion criteria of living with type 1 diabetes for at least one year, had not been diagnosed with any other chronic medical conditions, had been using closed-loop technology for at least three months, and had an HbA1c of < 7% and a TIR of > 70% (1, 2). Seven potential participants met the inclusion criteria and were invited via email to participate in online interviews. Five participants agreed to participate. Despite the relatively small sample size, numerous IPA studies have recruited approximately the same number of participants (33–36). Although the CDE is located in Johannesburg, South Africa, it caters to South Africans from all over South Africa, as it is a specialist centre for managing diabetes. Thus, participants were from different regions of South Africa.

### Research procedure and data collection

After receiving ethical approval from the Health Research Ethics Committee of the Faculty of Health Sciences, North-West University (NWU-00266-21-A1), a medical doctor at the CDE distributed an informational brochure to potential participants regarding the proposed study and invited them to participate in this research. Initial contact was made with the parents of the adolescents. Adolescents and their parents were provided with further information to make an informed decision regarding potential participation. It was emphasised that participation was voluntary and would not influence the care participants received at the CDE.

After participants completed the documents, the medical doctor directed them to the lead researcher. The project lead made initial contact with the potential participants and screened them. Potential participants were screened based on their age, the duration of their diabetes diagnosis, the presence of any other chronic conditions, their willingness to participate in online interviews, and whether their HbA1c level was <7% and their Time in Range (TIR) was >70%.

Participants’ answers to the screening questions determined whether they were eligible for this study. Those who did not qualify were given the option to receive a summary of the findings once this study was completed. The project lead emailed adolescent and parental consent forms to potential participants. Potential participants had two weeks to decide whether they wanted to participate. Before the interviews, the research procedure, goals, risks, benefits, and the voluntary nature of the study were thoroughly explained to the participants. Participants were notified that the interviews would be recorded and would only start after parental permission and adolescent consent had been obtained. Informed parental permission and adolescent consent were obtained from all participants. Parents’ involvement was limited to providing consent (verbal and written). They were provided with an explanation of the study and were provided with an opportunity to asks questions if they were uncertain about the process. Parents were not present during the interviews to ensure adolescents could speak freely and openly. This decision was made to avoid potential influence on the adolescents’ answers. The first author (SK), a registered psychologist, conducted open-ended interviews, each lasting approximately 30 to 45 minutes. The interviews were conducted online, in English (three interviews) and in Afrikaans (two interviews).

The interview schedule and prompting questions were developed in accordance with the research question and aim. The following questions were posed (1): Please tell me about yourself, (2)Please tell me more about the role of diabetes in your life, (3) Please tell me more about living with closed-loop technology, and (4) Is there anything else about your journey with diabetes that you would like to share today?

### Data analysis

After data collection, the interviews were transcribed by the first author (SK) and analysed (by SK and ED). The interview transcripts were analysed using the principles of IPA (25) to obtain an in-depth understanding of the adolescents’ adjustment experiences of living with well-controlled type 1 diabetes using closed-loop technology. The following steps were followed (25) (1): The researchers familiarised themselves with the data by reading and re-reading individual transcripts; (2) The transcripts were read line by line, and interpretative notes were made; (3) The researchers reviewed the transcripts and identified emerging themes. The identified themes were clustered and examined in relation to each other; (4) The researchers identified similarities across the emergent themes and created a list of superordinate themes. (Steps 1–4 were repeated for each transcript); and (5) A summary table was created as the final step in the analysis to identify themes across all transcripts. Superordinate themes were compiled from all the transcripts.

All identifying data were changed during the transcription phase. Anonymity was maintained during the research process by assigning a pseudonym to each participant. Participants’ pseudonyms were kept separate from their signed consent forms in order to prevent their identification. The voice recordings obtained during the in-depth interviews were transferred to the researcher’s (SK) and project lead’s (ED) password-protected computers and were deleted from the recording device. The transcriptions of the audio-recorded interviews reflected only the pseudonyms, and any identifiable data were thus eliminated.

The transcripts were referred to throughout the analysis to ensure the themes were grounded in the data. To ensure trustworthiness of the analysis, verbatim extracts reflecting the interpretations made were included while writing the study, as recommended by Smith et al. (25), and the research supervisors (ED, EvR and DS) reviewed all interpretations. The researchers maintained a reflective journal throughout the analytic process to ensure that the data represented participants’ true accounts. They referred to the transcripts throughout the analysis to ensure that the emerging themes supported the transcripts and were not biased. A co-coder (ED) was also used in this study. The co-coder is an experienced professor of psychology who has conducted numerous studies on diabetes in children, adolescents, and adults. The researchers who analysed the transcripts (SK and ED) compared their findings. Disagreements were resolved through discussion and consensus, which included all authors, with additional coding rounds as needed. This approach ensured rigorous and consistent theme development.

Verbatim quotes from the transcripts were included to support all the interpretations. To ensure transparency, during the analytic process and interpretation of findings, the research team engaged in reflective discussions to compare and reconcile individual interpretations of the data. When disagreements arose, we resolved them through collaborative discussions and re-evaluated the data. This consensus-building approach helped ensure the consistency and trustworthiness of the themes identified.

## Results

The sample comprised five participants, whose age at the time of the interviews and age at the time of diagnosis with diabetes are presented in **Table 1**. Pseudonyms are used to identify the participants from which illustrative extracts are used in the presentation of the findings below.

**Table 1:** Characteristics of the non-random purposive sample.

Pseudonym	Age	Age at diagnosis
Sophia	15	4
Olivia	18	5
Isabella	17	6
William	17	5
Charlotte	16	5

All participants indicated that their previous diabetes management included multiple daily injections, an independent insulin pump, and an insulin pump with a CGM.

The experiences of adjusting to closed-loop technology, as described by the group of adolescents living with well-controlled type 1 diabetes, were summarised by five superordinate themes identified through IPA (27): (1) learning to trust the technology, (2) making diabetes visible, (3) building a relationship with diabetes, (4) empowering support networks, and (5) transformative positive outcomes. The themes are summarised in **Table 2**.

**Table 2:** Themes Identified Through Interpretative Phenomenological Analysis.

Superordinate themes	Subordinate themes
**Learning to trust the technology**	Comparing closed-loop technology to multiple daily injections Initially struggling with trust
**Making diabetes visible**	Seeing leads to trust Seeing can lead to information overload and stress
**Building a relationship with diabetes**	Control leads to acceptance and the incorporation of diabetes into one’s identity The importance of routine and trial and error
**Empowering support networks**	Practical support Emotional support
**Transformative positive outcomes**	Improved quality of life Positive growth

### Learning to trust the technology

All participants’ accounts made it clear that trusting the closed-loop system was necessary to integrate the technology into their everyday lives. Two subthemes emerged: (1) comparing closed-loop technology to multiple daily injections and (2) initially struggling with trust.

#### Comparing closed-loop technology to multiple daily injections

All participants compared multiple daily injections and closed-loop technology, highlighting its strengths and weaknesses. Isabella (17 years old) asserted, ‘It was very difficult when I was injecting; it is much nicer with this pump.’ Olivia (18 years old) emphasised that it was ‘amazing … not having to constantly inject’.

Although all participants praised the pump for its ease of use, they mentioned that frequent alarms, problems with tubing, and having to calibrate their sensor were annoying and frustrating at times:

‘When the alarms constantly go off if you are trying to sleep …’ (Sophia, 15 years old)

‘It’s draining to literally have a machine attached to you 24/7.’ (Charlotte, 16 years old)

‘The pump makes my life so much easier, but sometimes you get needles that don’t work’. (Isabella, 17 years old)

Sophia’s (15 years old) frustration with intrusive alarms underscores the tension between the benefits of continuous monitoring and the intrusion into personal space and rest. This highlights adolescents’ balancing act when integrating technology into their lives. Charlotte’s (16 years old) sentiments reflect a profound emotional toll, indicating physical and psychological encumbrance. This opens a discussion on the need for psychological support structures for adolescents grappling with the constant presence of medical technology in their daily lives.

#### Initially struggling with trust

All participants referred to initially struggling to trust the technology. Olivia (18 years old) emphasised the importance of trusting the technology while indicating it was ‘a difficult adjustment to completely trust the machine’. She explained:

‘You can’t think for the technology that is already thinking for itself. Letting the machine think for me was a big adjustment’.

Olivia’s (18 years old) journey to relinquish control over technology encapsulates a key psychological adjustment, highlighting the trust involved in integrating technology into self-care routines. The research team interpreted this as a pivotal moment of transition, marking a shift in how adolescents perceive their role in managing diabetes.

Charlotte (16 years old) stated the following:

‘When I actually first got it [insulin pump], we didn’t trust it much. I used to wait and check everything and prick my finger because I was like, ‘Should I really trust my entire health with this little machine?’ But it is proven time and time again that it is perfect.’

Charlotte’s (16 years old) expression of trust reflects a common concern among adolescents regarding the reliability of medical technology. This hesitation illustrates the psychological hurdle of placing one’s health in the hands of technology. The researchers found this theme to recur, highlighting the nuanced process of building trust in medical devices.

For participants, seeing positive results (for example, the recommended HbA1c target) was a facilitator for trusting the technology once it had proven itself. Factors such as reduced HbA1c, fewer finger pricks, and automaticity contributed to trust in the technology. All participants praised the pump for reducing hypoglycaemia and warning them when their glucose levels rapidly decreased, which increased their trust in the technology.

Most participants controlled their diabetes well before using closed-loop technology and needed to adjust to trust that the pump would also manage their diabetes well. William (17 years old) said that he had realised that ‘technology is not perfect’. He indicated that, at times, he had experienced that the closed-loop system did not bring down his glucose levels enough, and then he would give a manual bolus when needed. Manual bolus refers to the manual administration of a specific amount of insulin by a user outside an automated closed-loop system. As reflected in participants’ narratives, trust was identified as a necessary component in adjusting to living with closed-loop technology. Participants had to adopt the belief that the technology could be trusted and that it would be beneficial for them to adjust to using closed-loop technology. The adolescents’ narratives highlighted an initial struggle with technology reliance, which pointed to a broader challenge of balancing autonomy and the desire for normalcy amid chronic illness management.

The transition from doubt to trust in the technology emerged as a pivotal theme. Participants narrated a journey of adaptation, characterised by an initial reluctance to relinquish control, followed by a gradual realisation of the technology’s reliability and the freedom it affords. This journey underscores the psychological adaptation necessary for accepting and integrating new diabetes management technologies, highlighting the importance of patience, openness to change, and the willingness to embrace new management paradigms.

### Making diabetes visible

Within this superordinate theme, two subthemes emerged: (1) seeing leads to trust, and (2) seeing can lead to information overload.

#### Seeing leads to trust

Observing and understanding their condition through real-time glucose data, often referred to by participants as ‘seeing’, transforms diabetes from an abstract concept into a tangible aspect of their lives they can manage and control. This ‘seeing’ refers to visualising diabetes management concretely through access to real-time glucose data. It increased trust and led to better diabetes management decisions, which emerged as an important part of the adjustment process. All participants referred to their ability to see the glucose trend graphs. They revealed polarised viewpoints, such as having constant access to glucose data and experiencing the benefits of obtaining a visual representation of diabetes. At the same time, some participants referred to ‘information overload’ in this regard.

All participants described the benefits of having access to continuous glucose data, emphasising convenience, enhancing feelings of safety, and experiencing a feeling of accomplishment when the glucose data were within the targeted range.

‘It’s taken a bit of worry off my shoulders that I’m able to monitor and see’. (Olivia, 18 years old)

‘So having a graph, it’s very convenient’. (William, 17 years old)

It’s so nice to look at my graph and be like, ‘Oh, I was 70% in range today.’ It’s just empowering. (Charlotte, 16 years)

William’s (17 years old) appreciation of graphical data highlights the empowerment derived from visualising one’s health data. The convenience he mentions transcends mere user-friendliness, touching on deeper themes of control and understanding one’s condition. This visualisation acts as a bridge between abstract numbers and tangible health-management strategies, thereby enhancing self-efficacy among adolescents. Overall, continuous feedback assisted participants in making more informed diabetes management decisions. Olivia (18 years old) described diabetes before closed-loop technology as a ‘guessing game’, and she praised closed-loop technology for its ability to visualise diabetes in a manner that demystifies the condition and empowers users to make informed management decisions. She remarked, ‘It really helped me stay in control because I knew the trends of what my blood sugar was doing’.

#### Seeing can lead to information overload and stress

While acknowledging that being able to ‘see’ diabetes – meaning having the ability to observe and interpret diabetes through detailed data visualisation – is a useful tool for assisting in making diabetes management decisions, some participants regarded it as information overload and a source of stress at times. Isabella compared multiple daily injections and closed-loop technology:

‘With injections, you almost forget that you have diabetes, but with the pump, you can always see the picture and always know what is going on, which is good, but it can also be stressful’.

Isabella’s (17 years old) comparison between injection and pump therapies illuminated the double-edged sword of continuous data visibility. Although the omnipresence of diabetes management data enhances control and self-awareness, it also imposes a constant reminder of the condition, leading to potential stress. This reflection sheds light on the psychological complexity of living with diabetes in the digital age, emphasising the need for strategies to manage not only the physical but also the emotional and cognitive burdens of such transparency.

William (17 years old) described the fact that diabetes can be unpredictable as a challenge:

‘On some days, you can do exactly the same, but your glucose levels will look different, and then you see it on the graph; it can be frustrating.’

Charlotte (16 years old) indicated that having the pump connected during all hours was ‘draining’. While all participants recognised the polarity of being able to see glucose data, they emphasised that it was a useful tool for adjusting to living with diabetes.

This superordinate theme indicated that it was empowering for participants to see the glucose data, where ‘seeing’ fundamentally means engaging with their diabetes through a visual and data-driven lens that makes management feel more controlled and less abstract. As Isabella (17 years old) described, being able to ‘see’ diabetes motivated her to manage her diabetes because when she could see a stable graph, she experienced a feeling of reward. Participants needed to adopt the perspective that obtaining a constant vision of diabetes would benefit them by adjusting to closed-loop technology. Throughout all the participants’ accounts, it became clear that establishing trust in the closed-loop system is essential for its integration into their everyday lives. This theme encompasses an initial scepticism followed by a gradual acceptance, marked by a nuanced understanding of the technology’s capabilities and limitations.

### Building a relationship with diabetes

A relationship with diabetes was built by engaging in two subthemes that dynamically interact over time: (1) control leads to acceptance and incorporation of diabetes into identity, and (2) the importance of routine, trial, and error. This process does not necessarily unfold in a linear manner but involves ongoing adjustments according to individual experiences and challenges.

#### Control leads to acceptance, normalisation and incorporating diabetes into identity

Incorporating closed-loop technology into their daily routines helped participants take control of their diabetes management, leading to a sense of acceptance, normalisation, and integration of diabetes into their identities.

Isabella (17 years old) gave the following explanation:

‘The more I started controlling diabetes, the easier it was to accept it and to realise that it will always be there … the pump helped me to accept diabetes, but the technology is just a catalyst to success; your attitude is important’.

William (17 years old) highlighted the importance of ‘discipline’ and ‘self-control’ in managing diabetes. He stated:

‘Diabetes teaches you discipline, because it’s so controlling, you know you can’t just do what you want, otherwise there will be consequences like a high sugar. For example, I would give myself so much insulin to correct the high I would go low, so it’s that discipline and self-control.’

For all participants, adjusting to closed-loop technology involved incorporating diabetes into their identities.

‘It does not make me who I am. It is part of you, but not who you are.’ (Isabella, 17 years old)

‘It doesn’t dehumanise you … it doesn’t make you not normal. You just have a few extra things that you have to do. It’s just one of those things; like some people, they wear glasses—it’s one of those things. (Olivia, 18 years old)

Charlotte (16 years old) indicated that she had initially embarked upon living with diabetes. She added:

‘Self-acceptance … I could fight it all I want; it’s not going away, it’s a part of me, it’s a personal choice how I want to deal with it … The only thing that can change is the way I deal with it. Life goes on with or without diabetes. Life goes on, and it’s really your choice how you want to monitor it’.

Sophia (15 years old) reported a similar experience and indicated that initially, she felt ashamed of living with diabetes, but then realised ‘I am so much more than diabetes’.

For Olivia (18 years old), closed-loop technology assists in experiencing a restored sense of self. She explained:

‘It made me feel more normal, in that I don’t have to constantly regulate, and I’ve also connected my CGM to my phone and I’m able to check it on my watch—my blood sugar—so I don’t have to constantly check and test all the time’.

Similarly, Sophia (15 years old) indicated that closed-loop technology ‘lets you live a more normal life’. Sophia’s statement, simple yet profound, speaks of the transformative potential of closed-loop technology for normalising the lives of adolescents with diabetes. As all participants have lived with diabetes for several years, they have likely incorporated a part of diabetes into their identity prior to using closed-loop technology. However, their engagement with closed-loop technology further transformed their relationship with diabetes. This suggests a regained sense of normalcy and independence, which the research team saw as the critical outcomes of successful technological integration. However, this relationship with diabetes is marked by tension, as illustrated by the analytic insights: while the technology aids in acceptance and normalisation, it simultaneously keeps the condition ever-present and visible.

The self-management routine led participants to view diabetes as manageable, and by taking responsibility for diabetes management, acceptance of diabetes was facilitated. Acceptance of diabetes led them to view diabetes as a part of who they were without negatively influencing their identities. Throughout all participants’ accounts, the psychological constructs of resilience and self-acceptance were evident, as all participants made closed-loop technology a part of their daily lives without technology taking over their lives. This adjustment process is dynamic and evolving, highlighting the complex interplay between technological innovation and personal growth in managing diabetes.

#### The importance of routine and trial and error

Several participants attributed their success to routine and a positive attitude. Olivia (18 years old) explained:

‘You can’t just expect the pump to give you insulin for food. You have to carb count, you have to bolus at a certain time before eating, but it’s adjustments that you have to make in order to get that HbA1c that you want. Success involves the technology, but your attitude, your determination, your hard work is what is eventually going to get you to that stage’.

It was evident that participants incorporated old self-management techniques and routines with the new technology and that it was a continuous process. Several participants referred to successful diabetes management as ‘trial and error’ by ‘finding what works for you’, having a ‘willingness to learn’ and routine. Olivia (18 years old) described diabetes as ‘second nature’. William (17 years old) also referred to the development of a routine: ‘I just go through the motions, you know, like I eat, give myself insulin; if it goes up, I correct’.

Participants emphasised that an essential part of adjusting to the technology was the realisation that diabetes could not be managed perfectly. They highlighted the importance of routine and the process of trial and error in navigating this adjustment, acknowledging that these elements are crucial for finding effective management strategies and accommodating the inherent imperfections of their condition.

‘Diabetes can’t be managed perfectly. I remind myself, I am a human being, I’m not a robot; my sugar isn’t going to be a perfect line; I am a human being. I have hormones; I have other things to take into account; I didn’t even realise how much everything impacts my sugar’. (Charlotte, 16 years old)

‘It’s not perfect … It happens and I correct and there is such a complex interaction of variables. It just happens, and then I try to deal with the aftermath’. (William, 17 years old)

In all participants’ accounts, the psychological factor of conscientiousness came to light, as all had the desire to manage their condition well and consistently applied their diabetes management routine, assisting in the process of adjusting to closed-loop technology. Building a relationship with diabetes reflects the fluid nature of managing diabetes, where control, acceptance and the establishment of a routine interact in a non-linear way, contributing to a comprehensive and personal understanding of living with diabetes.

### Empowering support networks

The importance of support stood out in the accounts of all participants. Support was identified at two levels: practical and emotional.

#### Practical support

Practical support included guidance from their doctors, training on the system, and hands-on assistance from their parents: Professional guidance from doctors and diabetes educators were instrumental in helping participants understand and use the closed-loop system effectively, as Olivia (18 years old) indicated: ‘My doctor helped me understand the pump.’This included troubleshooting advice, and ongoing consultations to ensure proper use of the technology. Comprehensive training on how to operate and manage the closed-loop system was essential. This training helped participants become familiar with the technology’s functions, setting adjustments, and troubleshooting common issues. Parents played a significant role in providing hands-on support, particularly in the earlier stages.

‘My mother, she knows everything, and she has all this knowledge. If I have a question, I can just ask her’. (William, 17 years old)

‘My parents were involved until Grade 8, helping me change my sites and reminding me when to do things. It is mostly me at this stage. My parents do give input, but it’s more suggestions, rather than them telling me what to do’. (Olivia, 18 years old)

This practical assistance not only made the technical aspects of diabetes management more manageable but also contributed to a smoother transition to greater independence in managing their condition.

#### Emotional support

All participants emphasised the value of emotional support in their journey to adjust to diabetes. Olivia (18 years old) noted, ‘How people say it takes a village to raise a child; it takes a village to raise a child with diabetes’.

Olivia’s analogy emphasises the indispensable role of a supportive community in the diabetes management journey. This highlights the multifaceted nature of support, which encompasses emotional, practical, and informational aspects. This communal approach not only aids in practical management but also in social and psychological adaptation to living with diabetes, underscoring the interdependence of individuals and their support systems in navigating chronic conditions. Support is critical for adjusting to living with diabetes. All participants emphasised the value of parental support.

‘We handle it as a team; as soon as I can talk to someone, I feel better’. (Sophia, 15 years old)

‘I wouldn’t have had the same HbA1c, I wouldn’t have been sitting here if I didn’t have support. I do not think I would have been alive if I did not receive any support. Support is the first thing to do’. (Olivia, 18 years old)

Some participants found social media helpful as a source of support:

‘I found on social media there’s a lot of groups and chatrooms that help, it’s other people giving you advice and also cheering you on’. (Olivia, 18 years old)

‘I think a big thing was just a diabetes support group and a diabetes support platform’. (Charlotte, 16 years old)

Sophia (15 years old) also referred to supportive siblings and revealed that being around her animals acted as a support system for diabetes-related distress:

‘My friends and my sister also help when I am going through an emotional time with diabetes. They encourage me to keep going, reminding me how far I have come, and also my parents; they are also always there. And then my dog ​​is just there, who gives a lot of attention and love when I need it’.

Olivia (18 years old) reported improved relationships because of closed-loop technology:

‘… giving my mum a lot of peace of mind because she’s also not stressing. It’s also improved our relationship because were not fighting so much about it’.

The closed-loop technology, by reducing the day-to-day burden of diabetes management, also lessened the emotional strain on both adolescents and their families, fostering a more supportive environment. Hence, emotional and practical support facilitate effective adjustment to living with type 1 diabetes. This significant reduction in stress for both Olivia and her mother highlights the profound impact of closed-loop technology on family dynamics. The decrease in daily tensions around diabetes management not only alleviates the emotional burden but also strengthens familial bonds. By reducing the frequency of conflicts related to diabetes care, closed-loop technology enhances the emotional well-being of those living with diabetes and their caregivers. Hence, the mutual reduction in stress contributes to a more harmonious living environment, underlining the importance of emotional and practical support in facilitating effective adjustment to living with type 1 diabetes. This example underscores the broader implications of advanced diabetes management technologies, improving individual health outcomes and enriching the quality of interpersonal relationships and overall family life.

The closed-loop technology not only empowered the participants by improving their diabetes management but also strengthened their support networks by fostering better communication, reducing stress, and enhancing the quality of relationships within their social and familial circles.

### Transformative positive outcomes

Participants reported two psychological factors as outcomes of successful adjustment: (1) improved quality of life and (2) positive growth.

#### Improved quality of life

The theme ‘Improved quality of life’ emerged as a significant finding in the experiences of adolescents who have successfully integrated closed-loop diabetes technology into their daily lives. Participants consistently highlighted how this technology has positively transformed their diabetes management, reducing the emotional and physical burdens associated with the condition.

For Olivia, the closed-loop system has provided a sense of security, especially during sleep, by alerting her to potential hypoglycemic events, thus reducing her fear of nighttime seizures. This sense of safety allows her to sleep more peacefully, which contributes significantly to her overall quality of life. Olivia (18 years old) said:

‘The technology will let me know if my blood sugar is going low. I can go to sleep at night knowing that I will hear my alarm going off if I’m going to seize; so, it’s not as big a fear as it was before the technology’.

Participants acknowledged that diabetes could take its toll emotionally at times; however, they all reported positive outcomes of diabetes that were part of successful adjustment to closed-loop technology. They reported an improved quality of life attributed to the closed-loop technology.

‘The technology has had a big influence and impact on my life and the quality of life that I have’. (Olivia, 18 years old)

‘It lets you live a normal life’. (Sophia, 15 years old)

‘… that extra level of comfort—knowing the alarm will wake me up and not having to stress.’ (William, 17 years old)

Sophia’s (15 years old) remark that the technology allows her to ‘live a normal life’ underscores the system’s ability to seamlessly integrate diabetes management into daily activities, minimising disruptions and allowing for a more typical adolescent experience. This normalcy is crucial during adolescence, a time when fitting in with peers and leading an active life are highly valued.

William’s (17 years old) comment about the ‘extra level of comfort’ from knowing that the alarm will wake him up if needed speaks to the reduced stress and anxiety associated with diabetes management. The reassurance provided by the closed-loop system helps him focus on other aspects of life without the constant worry of potential glucose-related emergencies.

Overall, the participants’ reflections illustrate how closed-loop technology has not only improved their physical health but also enhanced their emotional well-being, contributing to a better quality of life. The ability to manage diabetes with greater ease and confidence has allowed these adolescents to engage more fully in life, supporting their overall adjustment and fostering a positive outlook on living with diabetes.

#### Positive growth

Participants indicated a sense of gratitude, personal growth and an optimistic attitude:

‘It made me feel grateful because there are other diabetics who can’t afford this technology and who maybe don’t manage their diabetes well’. (Sophia, 15 years old)

‘It’s freeing, I can be a little bit more independent because I don’t have to worry how everything is going to affect my sugar, going to affect me. In a way, I am monitored’. (Charlotte, 16 years old)

As highlighted by Sophia’s comment, socio-economic status significantly influences access to and management of diabetes technology. The authors recognise that the ability of participants to afford advanced diabetes technology may reflect a higher socio-economic status compared to the average individual in South Africa. The disparity in access highlights a broader issue where those from higher socio-economic backgrounds may benefit from improved disease management options, while those from lower socio-economic backgrounds may face challenges that hinder their ability to achieve optimal diabetes control. This is an area that deserves further study.

‘I feel that it has helped me grow as a person: to be resilient. I take what I have and I use it to the best of my ability, and I am able to thrive with that, and especially the maturity it gave me’. (Olivia, 18 years old)

Olivia’s (18 years old) reflection on her personal growth because of managing diabetes using technology speaks to the transformative potential of living with a chronic condition. Her narrative transcends the day-to-day challenges of diabetes management to highlight broader psychosocial benefits, including resilience, maturity, and a proactive stance towards life challenges. This perspective illuminates the individuals’ capacity to find meaning and growth in their experiences of diabetes, suggesting a silver lining in the cloud of chronic conditions.

Charlotte (16 years old) elaborated on the need to assist others through her experiences of living with diabetes. According to William (17 years old), living with diabetes and closed-loop technology ‘makes you a stronger person’. Sophia (15 years old)indicated that living with diabetes leads to increased responsibility and maturity. She further revealed that she enjoyed educating others about diabetes and creating awareness of it.

The theme of transformative positive outcomes encompasses the psychological and quality-of-life enhancements attributed to successful adjustment to closed-loop technology. This theme captures the participants’ growth, resilience, and gratitude, offering a holistic view of the impact of technology on their lives. These narratives of positive transformation reflect a broad spectrum of benefits, from improved clinical outcomes to enhanced psychological well-being, illustrating the profound potential of technology to improve the lives of individuals with diabetes.

## Discussion

The narratives of this group of adolescents offer insights into their lived experiences of adjustment to closed-loop technology. Closed-loop technology positively impacted participants’ quality of life and diabetes self-management. Through the analysis, five superordinate themes were identified: (1) learning to trust technology, (2) making diabetes visible, (3) building a relationship with diabetes (4), empowering support networks, and (5) transformative positive outcomes. These themes demonstrate the adjustment process of adolescents to using closed-loop technology and align with the Unified Theory of Acceptance and Use of Technology (UTAUT) model. While an inductive approach, grounded in the data, was adopted in the design of the study and analysis of the data, some of our findings resonate with the propositions of the UTAUT, a widely used model in the field of technology (23). The UTAUT was used during the discussion phase to frame and interpret the findings. The UTAUT was initially developed to predict technology usage by individuals in general (23) and has also been adopted/utilised within the research field of medical devices and technologies, for example, Schretzlmaier et al. (24) explored the suitability of the UTAUT model in predicting the acceptance of mobile applications for diabetes management. Another example includes an investigation on the usage intentions of wearable medical devices using the UTAUT model (37). The study provided a demonstration of how UTAUT can be used to understand factors influencing the adoption of wearable technology in healthcare, particularly for continuous health monitoring. The UTAUT model has also been applied to study the adoption of electronic medical records (EMRs) by physicians (38).

According to the UTAUT model, effort expectancy, i.e. the ease of use of the technology, performance expectancy, i.e. how using the technology helps the individual attain improved performance, social influence, i.e. a person’s perception that significant others in their lives believe they should use the technology, and facilitating conditions, i.e. a person’s perception of how well the organisational and technical infrastructure supports the use of the technology are the most critical factors of behavioural intention (23, 39). The constructs of the UTAUT model, effort expectancy, performance expectancy, social influence, and facilitating conditions (6, 23), were reflected in participants’ accounts. Specifically, the ‘information overload’ theme reveals the complexity of ‘effort expectancy’ within this demographic. Adolescents’ accounts of managing constant data streams from their devices underscore the need for a balance between the ease of use of technology and the cognitive load it imposes. This extension of effort expectancy highlights the necessity for diabetes management technologies to be user-friendly and mitigate potential information overload, aligning with and expanding upon UTAUT’s considerations of technology adoption and usage. While the visibility of diabetes management data typically enhances performance expectancy, as adolescents perceive benefits in health management, our study adds to the UTAUT model by discussing how this visibility can also lead to information overload. This phenomenon complicates adolescents’ acceptance and use of technology, potentially overwhelming them with continuous data. By applying the UTAUT model to our findings, particularly the ‘information overload’ theme, we contribute to the literature by illustrating how the constant monitoring and data analysis required by closed-loop technology can influence adolescents’ psychological well-being and acceptance of the technology. This adds a layer to the model by suggesting that future research should consider the psychological impact of technology use beyond its practical applications, thereby extending the model’s applicability to health technologies that demand high user engagement.

All participants viewed closed-loop technology as relatively easy to use while acknowledging occasional hassles such as alarms and calibration, similar to the construct of effort expectancy. Overall, closed-loop technology was integrated into participants’ previous diabetes management. However, participants felt that in comparison to multiple daily injections, closed-loop systems were easier to use and had better management outcomes. A common response was that making closed-loop technology part of one’s daily routine was necessary for adjustment. In line with the construct of performance expectancy (23), participants viewed the closed-loop system as beneficial to their health and everyday life. They attributed the usefulness of the closed-loop system to the fact that glucose data were continuously available. They believed that the closed-loop system further enabled them to achieve improved glucose control and quality of life. They viewed the interaction with glucose information as empowering, a finding that coincides with a continuous glucose monitor (CGM) study indicating that access to glucose trends is empowering (40).

Previous studies suggest that not everyone benefits from closed-loop technology (41), but this finding contrasts with our study, where adolescents experienced improvements in Time in Range (TIR) and higher satisfaction with the technology (42). All participants in this study had well-controlled diabetes, likely contributing to a sense of mastery and positively influencing their adjustment process. The practical and emotional support from significant others was crucial in facilitating their adjustment, aligning with the social influence construct of the UTAUT model.

Several other facilitating conditions were identified, including making diabetes more tangible through a continuous visual representation of glucose data, making more informed management decisions, taking personal responsibility, positively integrating diabetes and closed-loop technology into one’s identity, and having a positive attitude. All participants emphasised the importance of routine. For all participants, it was essential to integrate diabetes and diabetes technology into their views. All participants were diagnosed at a relatively young age. Learning to live with diabetes from a young age may assist in the process of adjustment and creating a ‘new normal’ and it is possible that adolescents that have only been living with type 1 diabetes for a short period of time might have a different adjustment experience.

In addition to the UTAUT constructs, another construct reflected in participants’ accounts was trust in the closed-loop technology. Trust was a crucial component that facilitated adjustment to diabetes. This finding coincides with research indicating that trust is vital in accepting diabetes mobile applications (24) and that people using CGM often ascribe discontinuation due to a lack of trust in the technology (41). Research also indicates that trust increases confidence and reduces anxiety (43).

The above constructs were identified as crucial components in successful adjustment, but participants also referred to positive outcomes resulting from the adjustment process. All participants viewed diabetes as a manageable condition, with closed-loop technology as an important management tool and vehicle for successful adjustment. Positive attitudes were considered important in this study. Participants reflected on their personal growth, greater flexibility, positive emotions, and gratitude as outcomes of successful adjustment because of the closed-loop technology. The prior acceptance of other diabetes technologies, such as CGM, could have assisted in the transition to closed-loop technology, as all participants had used other diabetes technologies before using closed-loop technology. This prior acceptance and experience with diabetes technologies is an important characteristic of our sample and should be considered when evaluating the applicability and transferability of the results.

Previous research exploring psychological factors in individuals with type 1 diabetes has largely focused on maladaptive psychological factors (42). However, our study identified four interlinked psychological factors during the process of adjusting to closed-loop technology (1): perception, (2) attitude, (3) willingness to learn, and (4) motivation. Specifically, all participants perceived the closed-loop technology as helpful and trustworthy. They viewed the interaction with the visual glucose data as helpful and assisted them in making informed management decisions. Their perceptions of diabetes technology influenced their engagement with it. They viewed diabetes as a manageable condition and displayed an internal locus of control, as they took personal responsibility for managing the condition. The psychological factor of perception is in line with previous studies indicating that beliefs impact engaging with self-management behaviours (44, 45). According to participants’ accounts, an optimistic attitude was adopted, and the construct of gratitude was evident. Participants also demonstrated resilience and positive growth and reported that integrating diabetes and diabetes technology into their view of themselves (self-acceptance) was an important part of the adjustment process.

All participants displayed self-motivation and adopted the belief that managing diabetes was important; thus, they adopted a routine for managing their condition (habit formation). The sample of this study expressed their willingness to engage with technology and manage their condition. In building a relationship with diabetes, they displayed the personality traits of self-efficacy, conscientiousness, and positive affect. They were dedicated and committed to consistently applying their diabetes management routines, displaying self-discipline and control. This finding is consistent with research indicating that self-efficacy is associated with improved HbA1c (46) and a positive correlation between motivation and improved adherence (47). Most participants’ levels of training in diabetes self-management and technology were well-developed, which could influence their adjustment to the technology. All participants displayed a willingness to learn and engage with the technology. The willingness to learn and solve problems is an important factor in diabetes technology (48).

## Conclusion

The findings illustrate that adjustment is an ongoing process, and closed-loop technology is an important facilitator of successful adjustment. Adjustment is facilitated by being able to ‘see’ diabetes, which enables participants to make better diabetes management decisions and trust the technology, leading to improved diabetes management. Improved management and control of diabetes have led to the acceptance of diabetes technology and the incorporation of diabetes into their identity. Support is crucial throughout the adjustment process.

This study contributes by sharing the experiences of positive adjustment to closed-loop technology of a group of adolescents living with well-controlled type 1 diabetes. It provides information on the adjustment process and the psychological factors of perception, attitude, willingness to learn, and motivation and can, therefore, be used to inform interventions to assist others in adjusting to living with diabetes.

## Implications of the findings

The themes identified in this study describe the processes and outcomes of adjusting to closed-loop technology among adolescents living with type 1 diabetes. The impact of closed-loop technology goes beyond improved glucose control, and all participants reported an improved quality of life. While closed-loop systems have medical benefits, this study demonstrated that closed-loop technology might have psychological benefits and that psychological factors play a role in successful adjustment. Therefore, it is essential to focus on psychological factors when determining the optimal benefits of technology. This is an important finding which illustrates that while healthcare professionals should focus on the practical and medical aspects of closed-loop systems, emphasis should also be placed on psychological factors such as perception, attitude, willingness to learn, and motivation. The psychology of diabetes technology is poorly represented in literature and deserves further investigation. Even in this group of well-controlled adolescents, diabetes could not be perfectly managed. All participants acknowledged that diabetes management could be emotionally difficult at times, indicating the importance of a holistic approach. Moreover, they emphasised the importance of support for successful adjustment, which should be an area for further research to determine the best way to support people living with diabetes.

It is evident that closed-loop technology is a tool for successful diabetes management and adjustment; however, as highlighted by all participants, the engagement and management of the person living with diabetes are crucial. Design improvements in closed-loop technology could include customisable alerts catering to adolescents’ unique interaction patterns. In conclusion, interventions should not only focus on the practicalities of managing diabetes and using closed-loop technology but also consider the psychology of successful adjustment. Medical treatments for adolescents with type 1 diabetes can be enhanced by incorporating closed-loop technology tailored to the unique challenges and lifestyles of this age group. This includes developing treatment plans that account for the variability in adolescent schedules, activities, and emotional states.

A differentiated approach should be taken to supporting adolescents and adults using diabetes technologies. This involves recognising the distinct ways in which adolescents interact with technology and their social environments and how peer influence plays a role in technology acceptance and sustained use. Educational programmes tailored to adolescents should leverage social media and peer support to enhance engagement and adherence.

## Future research directions

Psychological factors play an important role in adjusting to closed-loop technology. Although useful insights could be gained from existing qualitative research exploring patient engagement with home use medical technologies, no other studies could be found which explore specifically adolescents’ lived experiences of adjusting to closed-loop technology, a gap that the present study sought to address. Future research could build on and expand these findings by exploring the adjustment to closed-loop technology in a broader range of settings and populations. This includes multicentre studies that capture diverse experiences and potentially different outcomes. Future research could consider including parental input in addition to the lived experiences of adolescents.

Further research should explore the longitudinal impacts of technology use on adolescents’ diabetes management and quality of life to provide deeper insights into the long-term benefits and challenges of closed-loop systems. A particularly valuable direction for future research would be to investigate the experiences of adolescents who do not perceive closed-loop technology as beneficial in managing their diabetes. Such studies could uncover critical factors that limit the technology’s efficacy, offering perspectives on when and why closed-loop technology may not fulfil its therapeutic potential. The findings lay the foundation for possible interventions and other studies within the context of psychological factors and adjustments to closed-loop technology.

## Data Availability

The raw data supporting the conclusions of this article will be made available by the authors, without undue reservation.
